# Therapy resistance on the RADar in ovarian cancer

**DOI:** 10.15252/emmm.202114010

**Published:** 2021-03-29

**Authors:** Jonas Schwickert, Franziska M Zickgraf, Martin R Sprick

**Affiliations:** ^1^ Division of Stem Cells and Cancer German Cancer Research Center (DKFZ) Heidelberg Germany; ^2^ Heidelberg Institute for Stem Cell Technology and Experimental Medicine (HI‐STEM gGmbH) Heidelberg Germany; ^3^ Faculty of Biosciences Heidelberg University Heidelberg Germany

**Keywords:** Biomarkers & Diagnostic Imaging, Cancer

## Abstract

Ovarian cancer has the worst prognosis of all gynecological cancers with high‐grade serous ovarian cancer (HGSOC) accounting for the majority of ovarian cancer deaths. Therapy resistance and the selection of effective therapies for patients remains a major challenge. In this issue of *EMBO Molecular Medicine*, Hoppe *et al* present RAD51 expression as a biomarker of platinum resistance in high‐grade serous ovarian cancer (HGSOC) patients (Hoppe *et al*, 2021).

## Graphical abstract

M. Sprick, J. Schwickert and F. Zickgraf discuss the identification of RAD51 as a biomarker of platinum resistance in High‐Grade Serous Ovarian Cancer patients, as reported by A. Jeyasekharan and colleagues in this issue of *EMBO Molecular Medicine*.

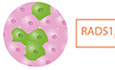

The 5‐year overall survival rate of HGSOC is < 50% and drops as low as 29% when it has metastasized to distant organs (Siegel *et al,* 2020). The standard‐of‐care treatment for HGSOC is a combination of debulking surgery followed by a platinum‐based chemotherapy together with paclitaxel. However, the majority (> 80%) of patients face relapse due to therapy resistance.

Recurrence of ovarian cancer is staged as either platinum‐sensitive (platinum‐free interval (PFI) > 6 months) or platinum‐resistant (PFI < 6 months) accompanied by poor survival (Matulonis *et al,* 2016). So far, no clinical biomarkers for platinum resistance have been approved (Hoppe *et al,* 2018).

The sensitivity of ovarian cancers for platinum‐based therapy can be explained in part by homologous recombination repair (HRR) pathway deficiency (HRD). In total, more than 50% of ovarian cancers show defects in HRR pathway genes including the *BRCA*/Fanconi Anemia network (Cancer Genome Atlas Research, 2011). Well‐known members of HRR pathway are BRCA1 and BRCA2, as well as RAD51, which is loaded onto the DNA by BRCA2. RAD51 acts as a central recombinase in HR that is important for homology search and strand exchange (Baumann & West, 1998). Hoppe *et al* (2021) now demonstrate that RAD51 overexpression correlates with platinum resistance in a subset of HGSOC patients.

The authors developed a quantitative immunohistochemistry (qIHC) assay to analyze RAD51 expression levels in formalin‐fixed paraffin‐embedded (FFPE) tissues. They defined a RAD51 nuclear expression score (RAD51_NES_) using the average expression of RAD51 across all imaged tumor cells measured by qIHC. They tested the clinical relevance of RAD51_NES_ on the British Columbia Cancer (BCC) Vancouver cohort treated with standard‐of‐care therapy. RAD51_NES_ followed a normal distribution and could be used as a categorical variable dividing the patient cohort into low and high RAD51_NES_. Of note, they could show that high RAD51_NES_ was associated with reduced progression‐free survival (PFS) and overall survival (OS), suggesting a higher risk for platinum resistance (Fig 1).

To exclude the potential effects of the taxane component used in the standard‐of‐care therapy in the BCC cohort, the authors further validated their findings in the SCOTROC4 cohort, which is composed of patients in a carboplatin monotherapy trial (Stronach *et al,* 2018).

In this cohort, neither the absolute HRD scores nor *BRCA* mutations were associated with RAD51_NES_. Thus, the authors suggest that the mechanisms that drive RAD51 expression in ovarian cancer are independent from a recombination defect. However, when the patients were subdivided according to their HRD status, the RAD51_NES_ could be used to predict patient survival within the HRD‐negative subgroup but not within the HRD‐positive subgroup. In the HRD‐negative subgroup, RAD51_NES_‐low patients showed an increased PFS and OS compared to RAD51_NES_‐high patients, suggesting that RAD51 expression predicts platinum resistance mostly in patients with intact HR.

As the authors discuss, a “cut‐off” for the RAD51_NES_ has to be defined and validated in further prospective studies to make this score suitable for the clinical use. Equally important is the validation in a cohort treated with the standard‐of‐care protocol based on platinum salts in combination with taxanes, as the RAD51_NES_ has so far only been validated in a platinum monotherapy cohort for the ability to stratify HRD‐negative HGSOC patients.

Intriguingly, overexpression of RAD51 in HGSOC cell lines did not result in increased platinum resistance. Therefore, the authors performed transcriptomic analysis on these cell lines and identified an enrichment in genes related to T‐cell‐mediated and B‐cell‐mediated immunity, suggesting a potential role of tumor‐immune interactions in platinum‐resistance in patients.

Thus, the authors analyzed the tumor microenvironment of RAD51_NES_‐high patient samples at a single‐cell resolution using multispectral qIHC, focusing on T cells and macrophages. They observed a significant exclusion of CD3^+^/CD8^+^ cytotoxic T cells from tumor regions in RAD51_NES_‐high cancers in the BCC cohort. A less prominent effect was also observed in CD3^+^/FOXP3^+^ regulatory T cells, but not in macrophages.

The authors speculate that high levels of RAD51 promote a yet unknown immune checkpoint preventing T‐cell infiltration into the tumor. While further studies on this subject are necessary, such findings could result in possible therapeutic interventions in RAD51_NES_‐high tumors. For example, immune checkpoint inhibitors could be administered to patients with RAD51_NES_‐high tumors to promote anti‐tumor immunity. Of note though, no successful immunotherapy for ovarian cancer has been identified so far (Kandalaft *et al,* 2019).

In addition to its significance for platinum‐based therapy, RAD51_NES_ might also be a potential biomarker stratifying the clinical outcome for patients treated with another class of therapeutic agents, such as PARP inhibitors, which are synthetic lethal in combination with a mutation in BRCA1/2 and have been shown to be highly efficient in HGSOC patients sensitive to platinum therapy (Lord & Ashworth, 2017).

With the RAD51_NES_, Hoppe and colleagues present a promising biomarker for platinum resistance that can help to better stratify HDR‐negative HGSOC patients, with the potential to improve the therapy of this devastating cancer.

**Figure 1:**
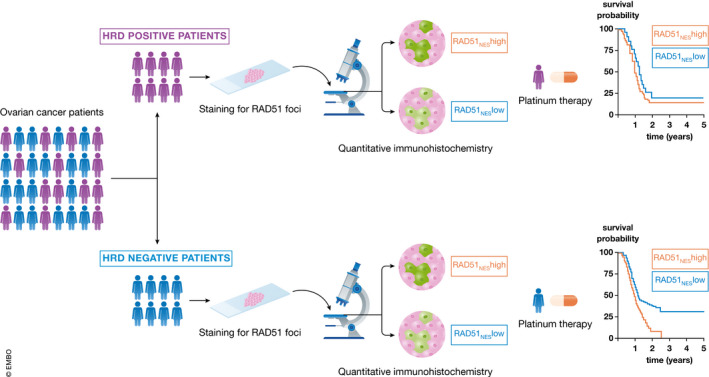
Ovarian cancer patients can be subdivided into two groups based on their HRD status By defining the RAD51_NES,_ it is possible to stratify HRD‐negative patients undergoing platinum‐based therapy for clinical outcome.
